# Supplementary figures and images for: Small Extracellular Vesicles From Radioresistant H3K27M‐Pediatric Diffuse Midline Glioma Cells Modulate Tumor Phenotypes and Radiation Response
Source: J Extracell Vesicles. 2025 Oct 30;14(11):e70188. doi: 10.1002/jev2.70188 (PMC12575060; doi:10.1002/jev2.70188)

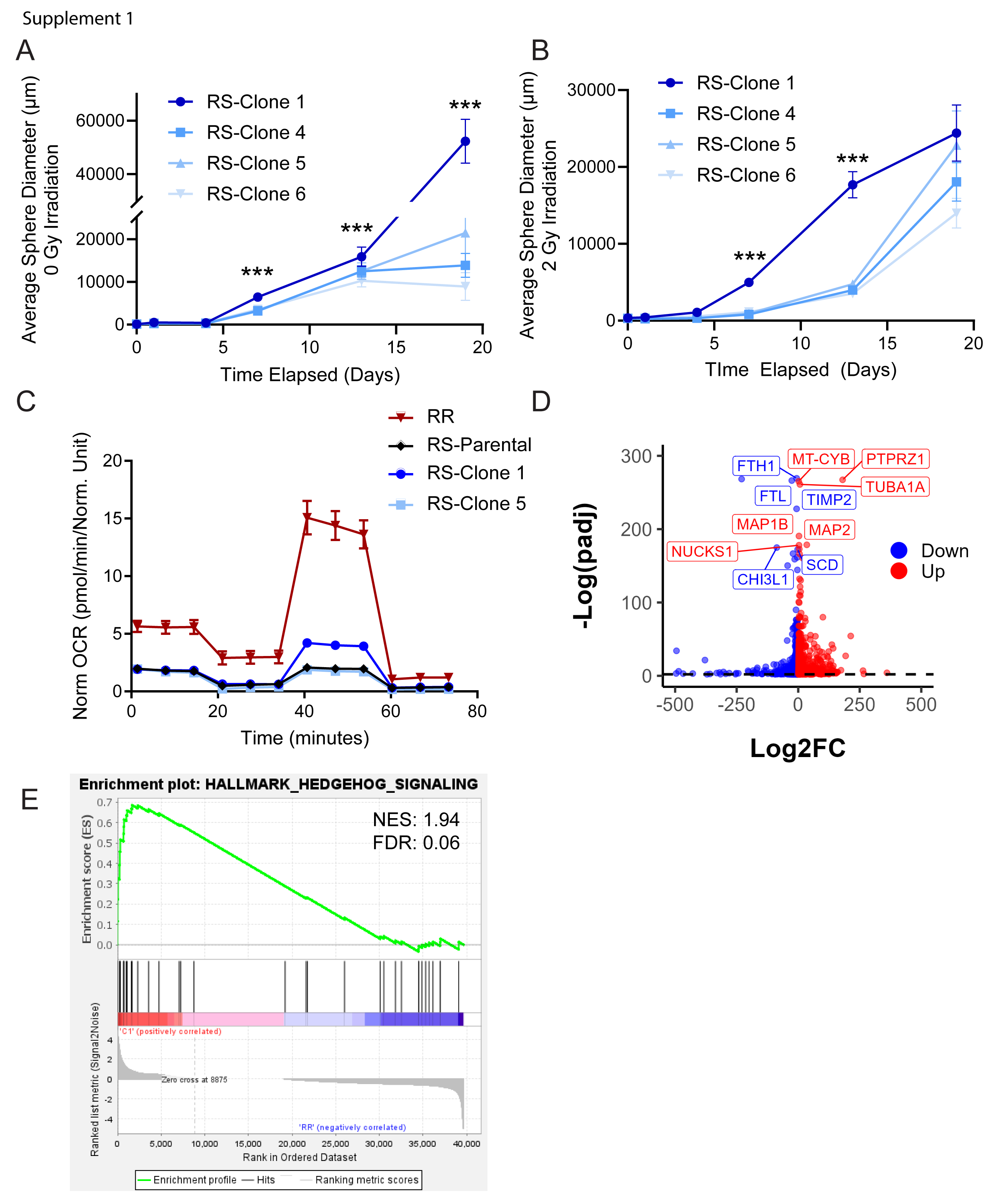

Supplement: Supplementary file 1 — Supplementary Figure 1: jev270188‐sup‐0001‐FigureS1.tif [file JEV2-14-e70188-s003.tif]

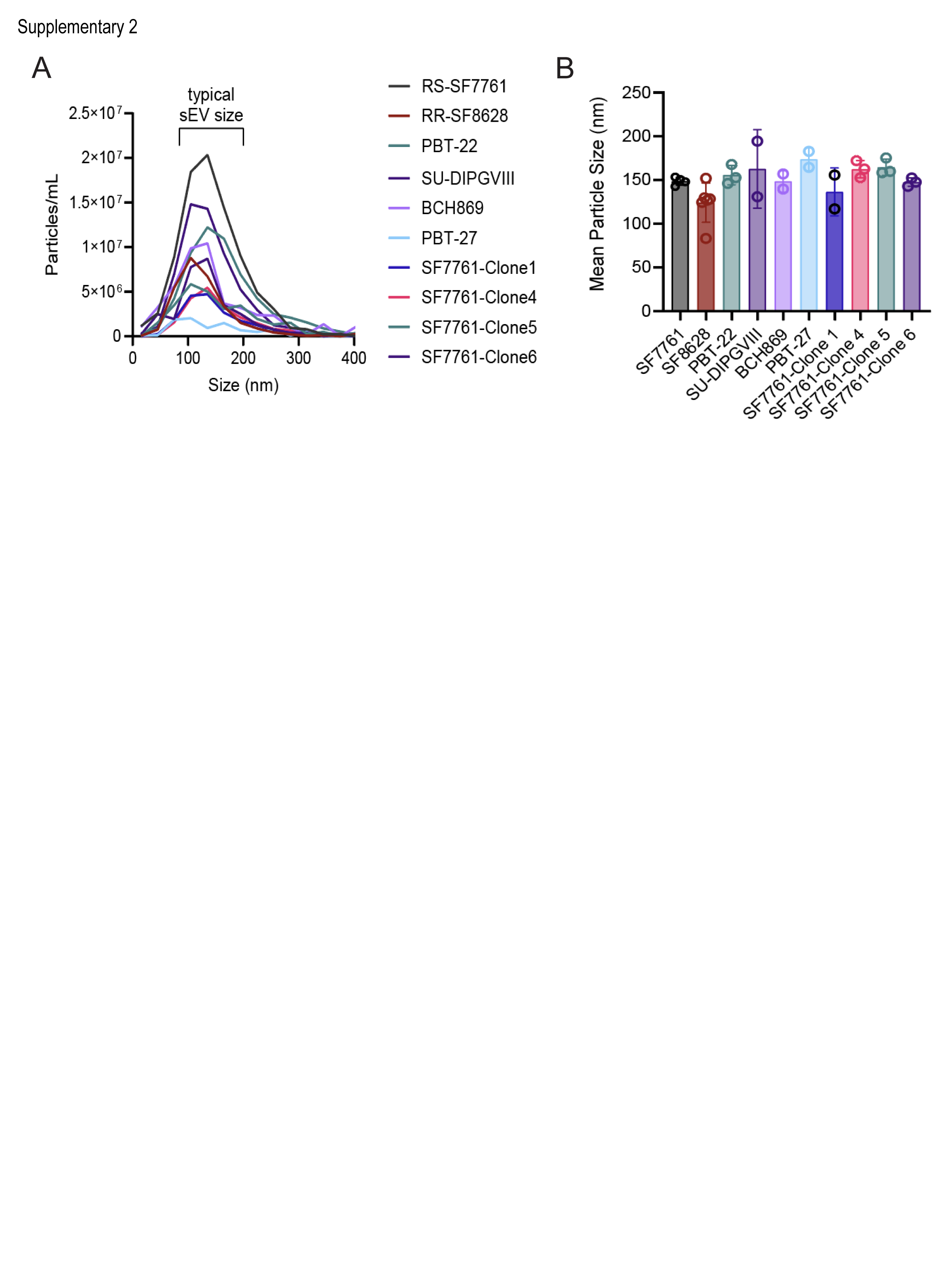

Supplement: Supplementary file 2 — Supplementary Figure 2: jev270188‐sup‐0002‐FigureS2.tif [file JEV2-14-e70188-s012.tif]

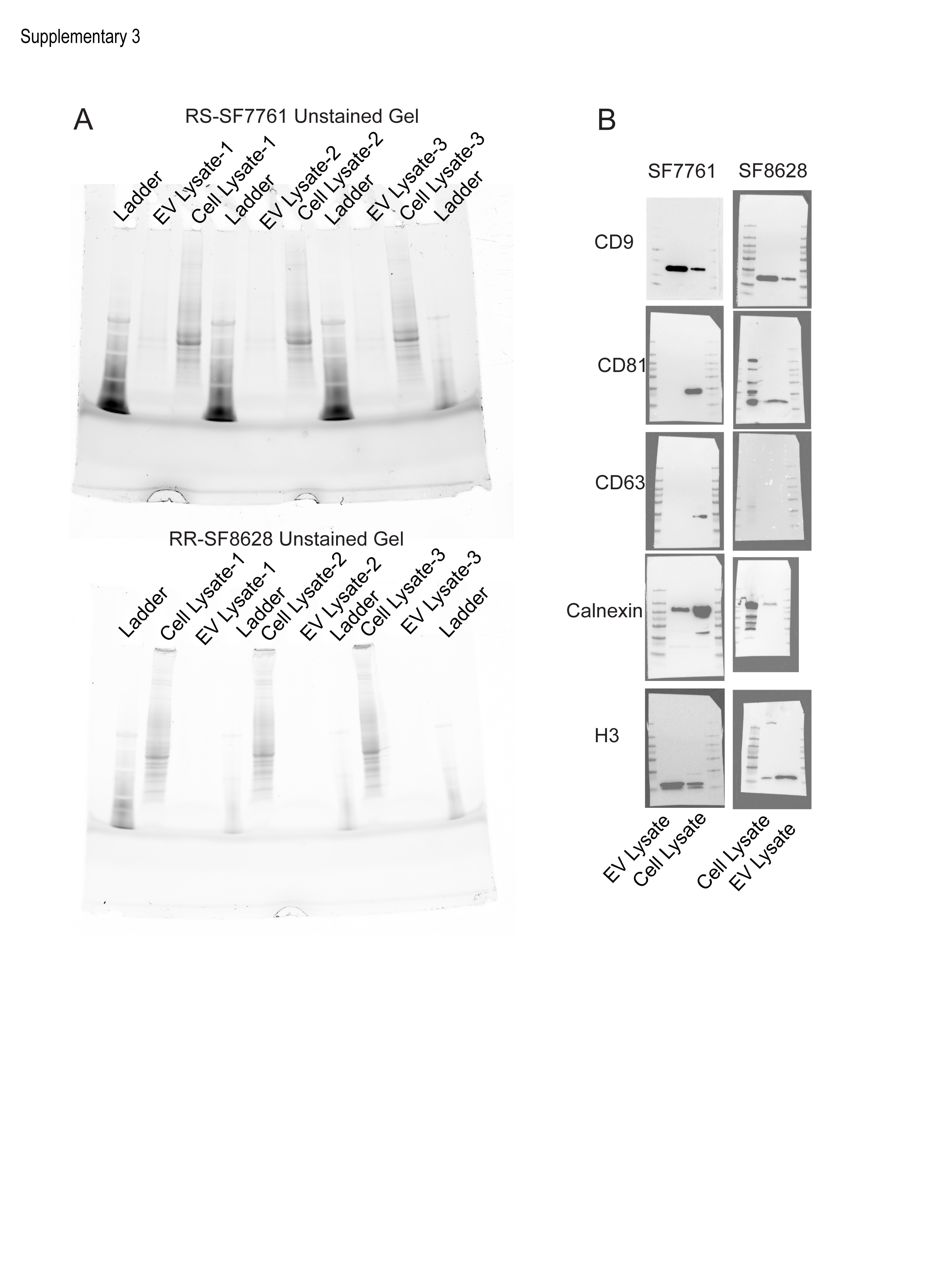

Supplement: Supplementary file 3 — Supplementary Figure 3: jev270188‐sup‐0003‐FigureS3.tif [file JEV2-14-e70188-s013.tif]

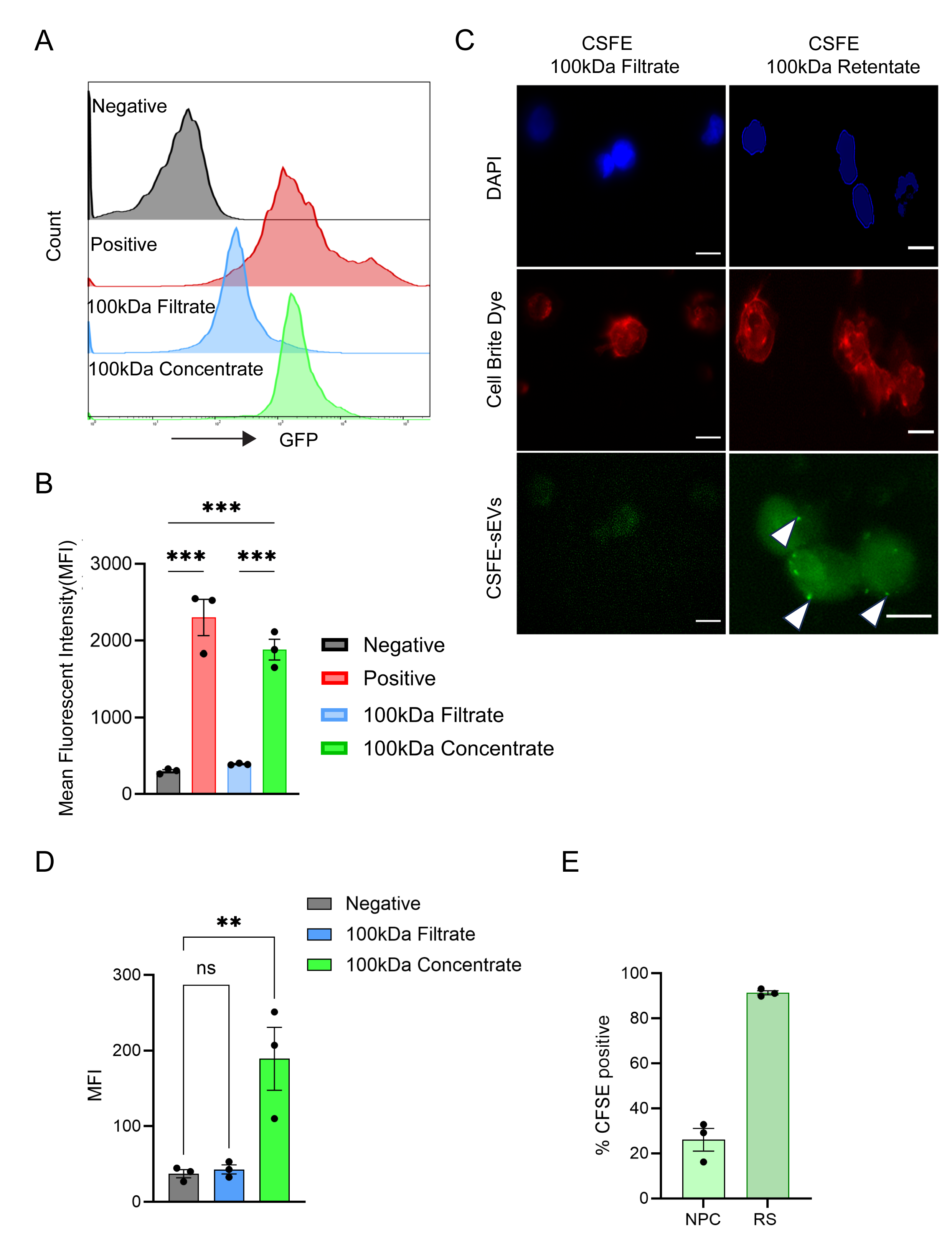

Supplement: Supplementary file 4 — Supplementary Figure 4: jev270188‐sup‐0004‐FigureS4.tif [file JEV2-14-e70188-s007.tif]

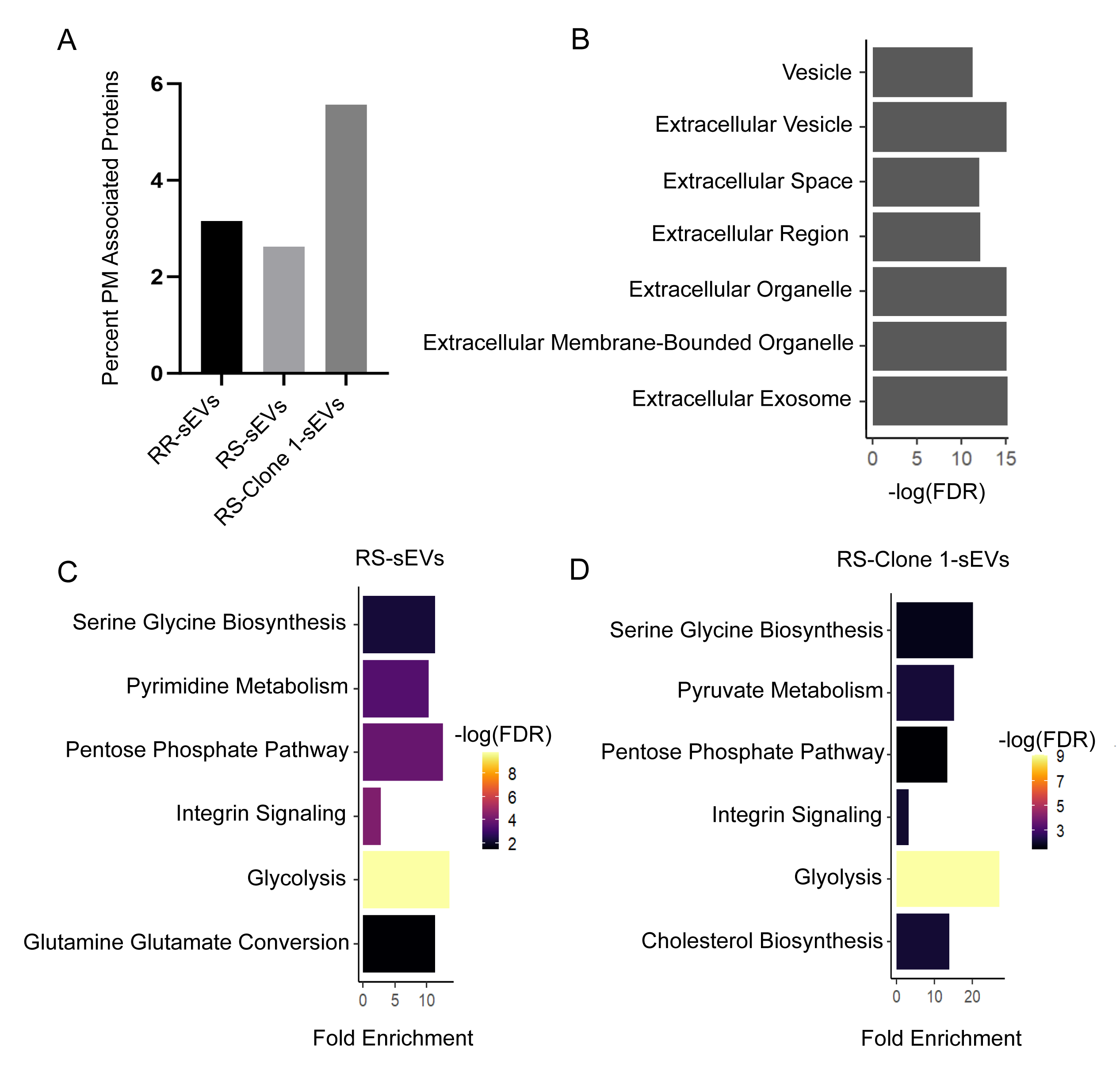

Supplement: Supplementary file 5 — Supplementary Figure 5: jev270188‐sup‐0005‐FigureS5.tif [file JEV2-14-e70188-s005.tif]

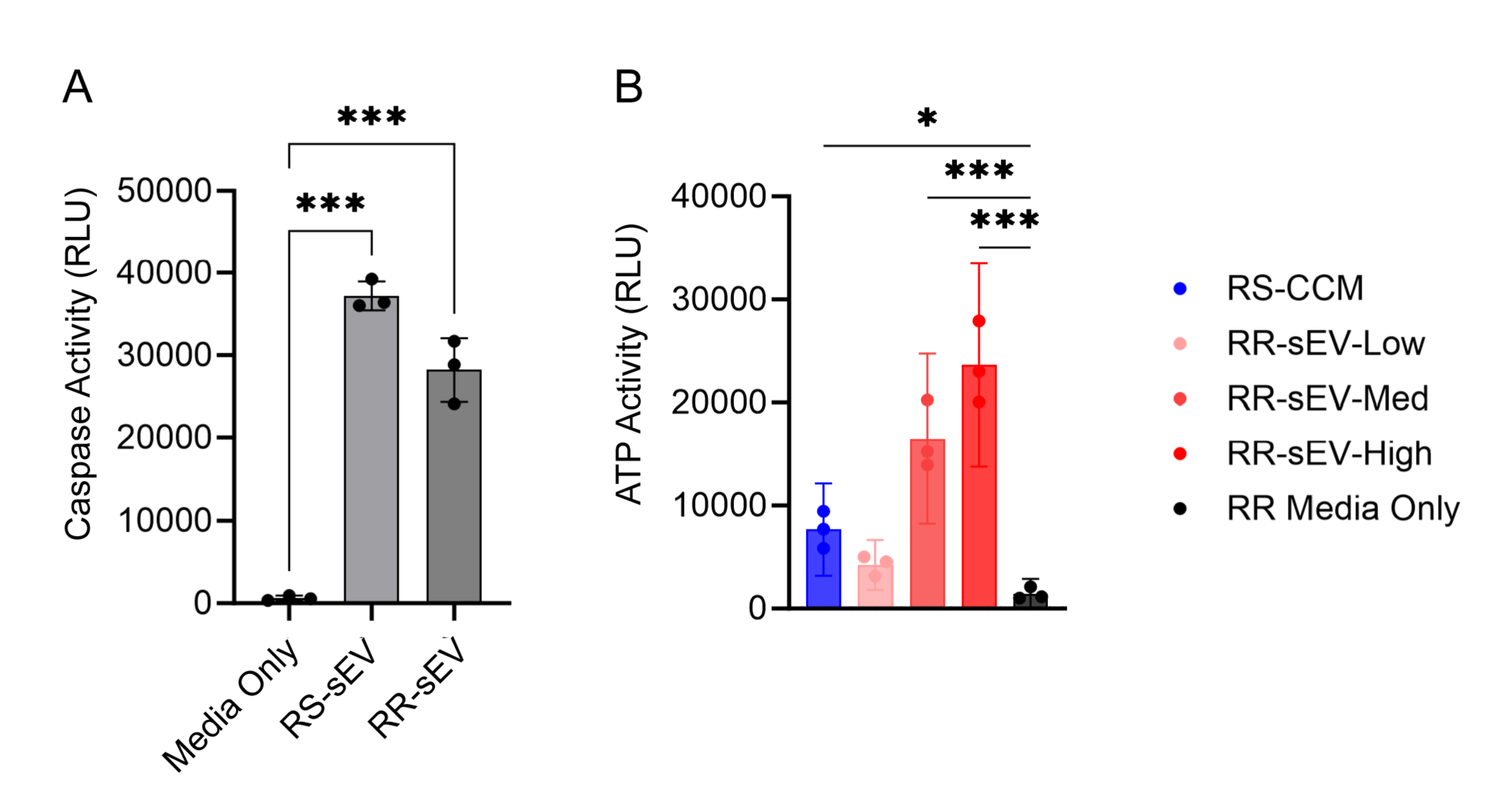

Supplement: Supplementary file 6 — Supplementary Figure 6: jev270188‐sup‐0006‐FigureS6.tif [file JEV2-14-e70188-s001.tif]

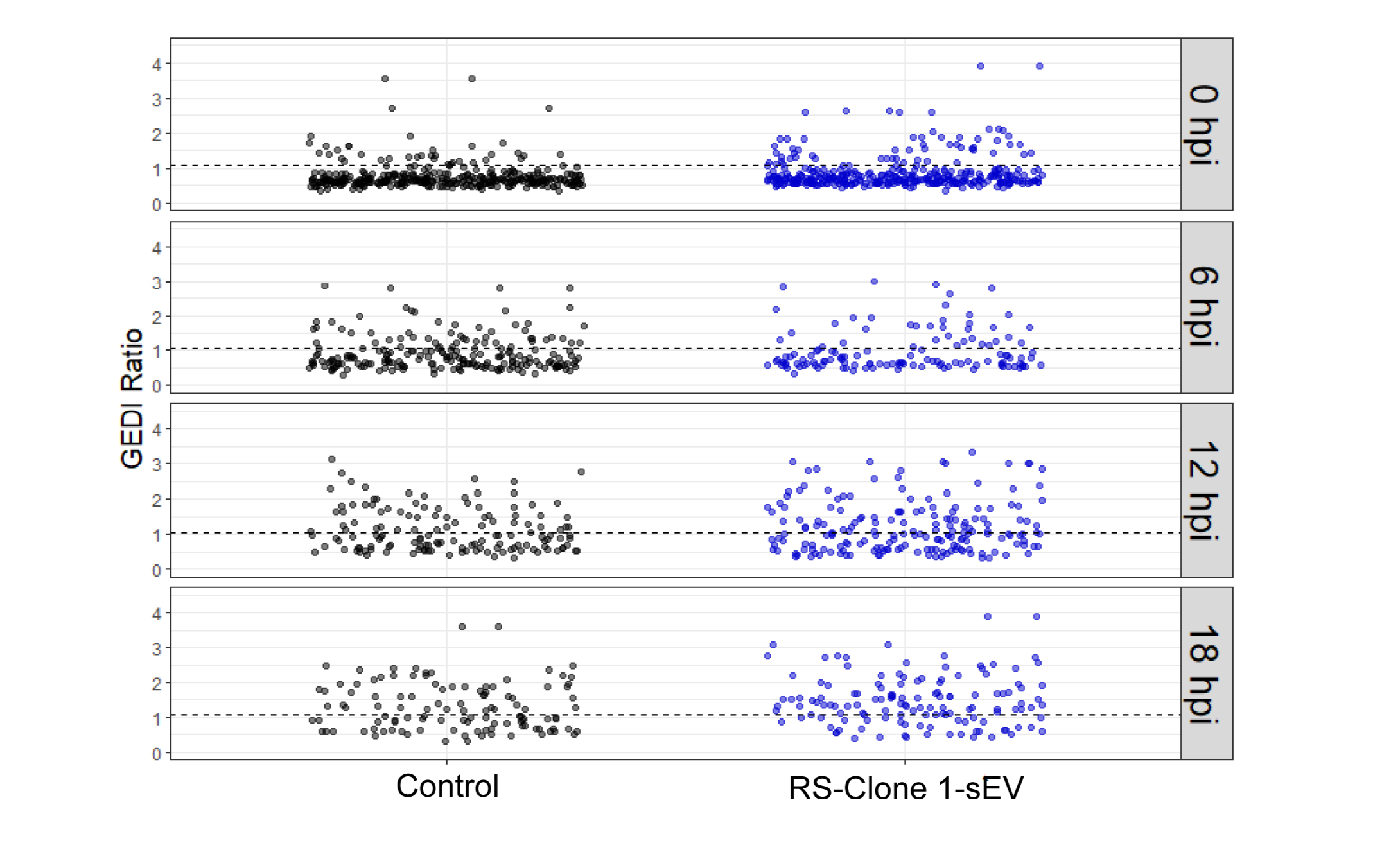

Supplement: Supplementary file 7 — Supplementary Figure 7: jev270188‐sup‐0007‐FigureS7.tif [file JEV2-14-e70188-s006.tif]

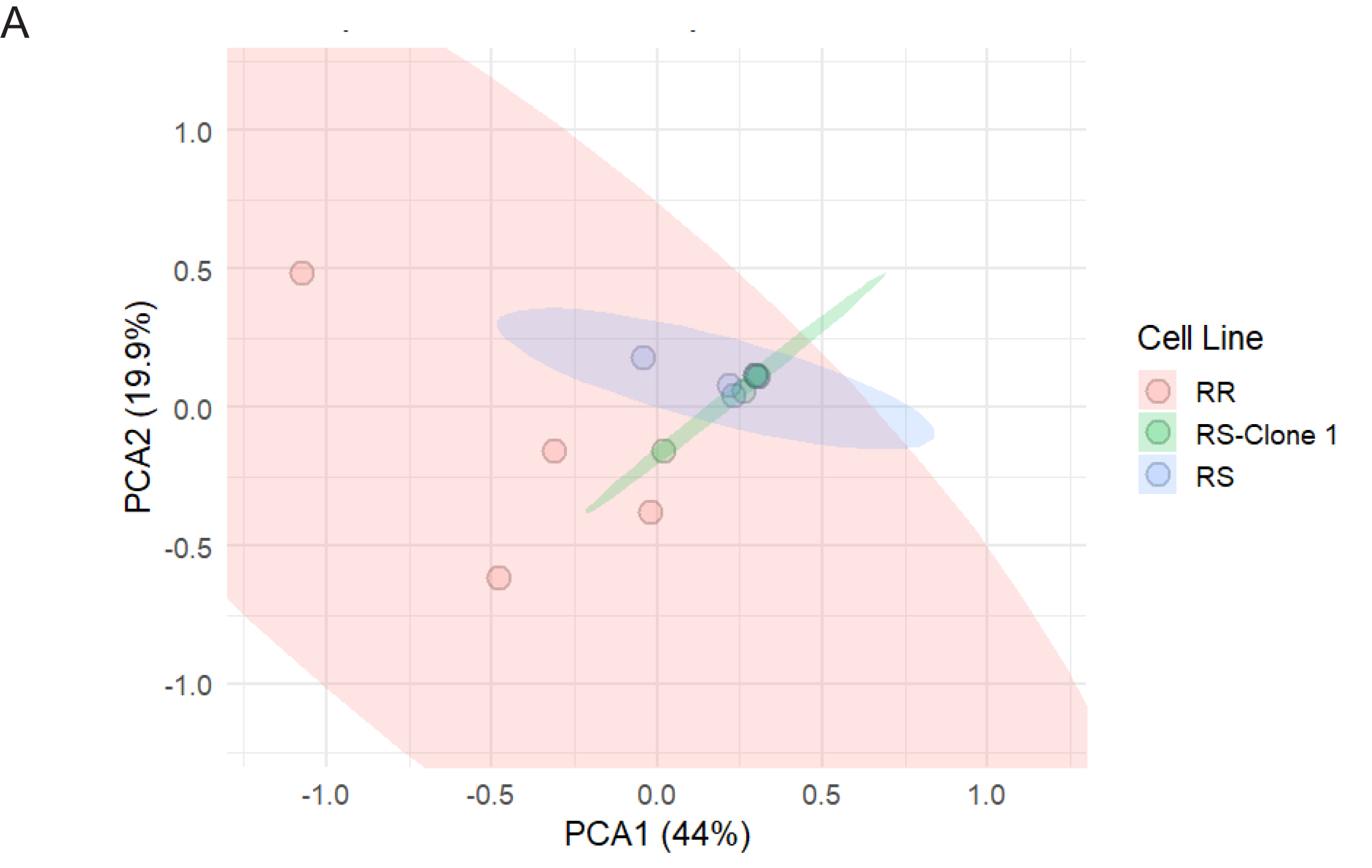

Supplement: Supplementary file 8 — Supplementary Figure 8: jev270188‐sup‐0008‐FigureS8.tif [file JEV2-14-e70188-s009.tif]

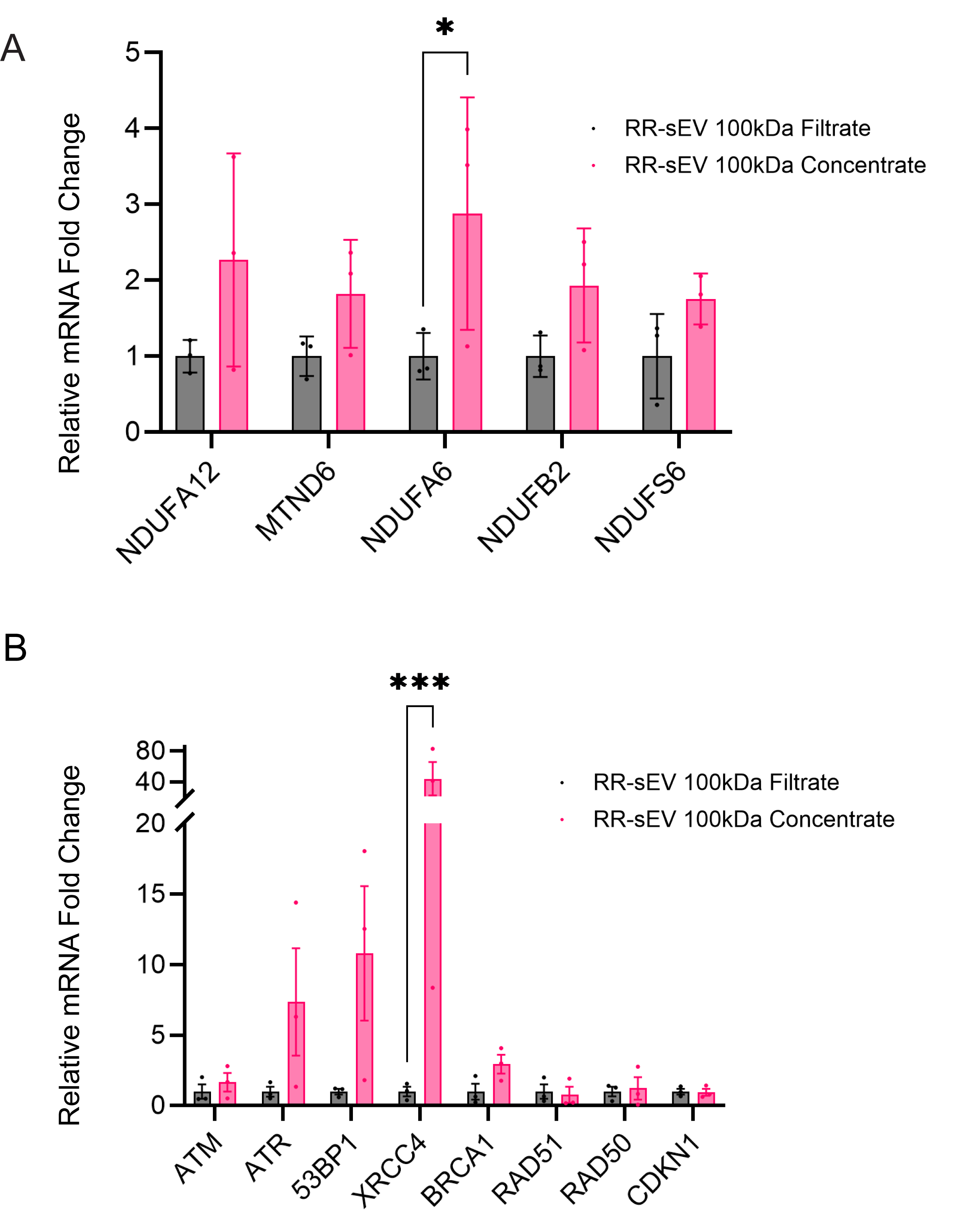

Supplement: Supplementary file 9 — Supplementary Figure 9: jev270188‐sup‐0009‐FigureS9.tif [file JEV2-14-e70188-s004.tif]
