# Supplementary material for: Small Extracellular Vesicles From Radioresistant H3K27M‐Pediatric Diffuse Midline Glioma Cells Modulate Tumor Phenotypes and Radiation Response
Source: J Extracell Vesicles. 2025 Oct 30;14(11):e70188. doi: 10.1002/jev2.70188 (PMC12575060; doi:10.1002/jev2.70188)
Supplement: Supplementary file 13 — Supplementary Table 5: jev270188‐sup‐0013‐TableS5.docx [file JEV2-14-e70188-s010.docx]

**Supplementary Table 5: H3K27M-pDMG extracellular vesicle MISEV2023 protein content markers**

| **Category** | **Cell Line** | | **Proteins** | **Confidence of Identification** | **Number of Samples identified in** | |
| --- | --- | --- | --- | --- | --- | --- |
| **Presence of EV features (Category 1 and 2)** | | | | | | |
| Transmembrane proteins | SF8628-RR | | GNA*, ITG*, | High | 3/3 | |
| Transmembrane proteins | SF7761-RS-Clone 1 | | ITG*, GNA*, BSG, CD81, LAMP2, ADAM10, NT5E,CD47, CD9, CD82, CD63 | Low to High | 2/3 | |
| Transmembrane proteins | SF7761-RS | | ITG*, GNA*, BSG, CD81, LAMP2, ADAM10, NT5E, CD47, CD9, CD82, CD63 | Low to High | 3/3 | |
| Cytosolic proteins | SF8628-RR | | HSPA8, CAV*, ACT*, GAPDH, TUB* | High | 3/3 | |
| Cytosolic proteins | SF7761-RS-Clone 1 | | SDCBP, GAPDH, ACT*, HSPA8, TUB*, FLOT2, TSG101, VPS4B | Low to High | (1-3)/3 | |
| Cytosolic proteins | SF7761-RS | | ACT*, TUB*, GAPDH, HSPA8, GNA*, FLOT1/2, TSG101 , VPS4B | Low to High | (1-3)/3 | |
| **Purity Assessment (Category 3)** | | | | | | |
| Lipoproteins | SF8628-RR | | n/a | n/a | n/a | |
| Lipoproteins | SF7761-RS-Clone 1 | | n/a | n/a | n/a | |
| Lipoproteins | SF7761-RS | | n/a | n/a | n/a | |
| Protein/nucleic acid aggregates | SF8628-RR | | YWHAZ | High | 3/3 | |
| Protein/nucleic acid aggregates | SF7761-RS-Clone 1 | | not found | not found | not found | |
| Protein/nucleic acid aggregates | SF7761-RS | | not found | not found | not found | |
| Exomere/supermere enriched | SF8628-RR | | HSP90AA/AB1, LDHA | High | 3/3 | |
| Exomere/supermere enriched | SF7761-RS-Clone 1 | | not found | not found | not found | |
| Exomere/supermere enriched | SF7761-RS | | not found | not found | not found | |
| **Intracellular EV origin (Category 4 and 5)** | | | | | | |
| Nucleus | | SF8628-RR | not found | not found | | not found |
| Nucleus | | SF7761-RS-Clone 1 | not found | not found | | not found |
| Nucleus | | SF7761-RS | not found | not found | | not found |
| Mitochondria | | SF8628-RR | not found | not found | | not found |
| Mitochondria | | SF7761-RS-Clone 1 | not found | not found | | not found |
| Mitochondria | | SF7761-RS | not found | not found | | not found |
| Secretory pathway | | SF8628-RR | not found | not found | | not found |
| Secretory pathway | | SF7761-RS-Clone 1 | HSPA5, HSP90B1, CANX | Low-High | | (1-3)/3 |
| Secretory pathway | | SF7761-RS | HSPA5, HSP90B1, CANX | High | | 3/3 |
| Cytokines/Growth factors | | SF8628-RR | not found | not found | | not found |
| Cytokines/Growth factors | | SF7761-RS-Clone 1 | EGFR, IL*, FGF2 | Low-High | | (1-3)/3 |
| Cytokines/Growth factors | | SF7761-RS | EGFR, IL*, FGF2 | High | | 3/3 |
| Adhesion/ECM | | SF8628-RR | COL** | High | | 3/3 |
| Adhesion/ECM | | SF7761-RS-Clone 1 | LGALS3BP, MFGE8, AHSG, FN1 | Low-High | | (1-3)/3 |
| Adhesion/ECM | | SF7761-RS | LGALS3BP, MFGE8, AHSG, FN1 | High | | 3/3 |
| Other | | SF8628-RR | not found | not found | | not found |
| Other | | SF7761-RS-Clone 1 | not found | not found | | not found |
| Other | | SF7761-RS | not found | not found | | not found |
