# Supplementary material for: Small Extracellular Vesicles From Radioresistant H3K27M‐Pediatric Diffuse Midline Glioma Cells Modulate Tumor Phenotypes and Radiation Response
Source: J Extracell Vesicles. 2025 Oct 30;14(11):e70188. doi: 10.1002/jev2.70188 (PMC12575060; doi:10.1002/jev2.70188)
Supplement: Supplementary file 15 — Supplementary Materials: jev270188‐sup‐0006‐Figures.docx [file JEV2-14-e70188-s008.docx]

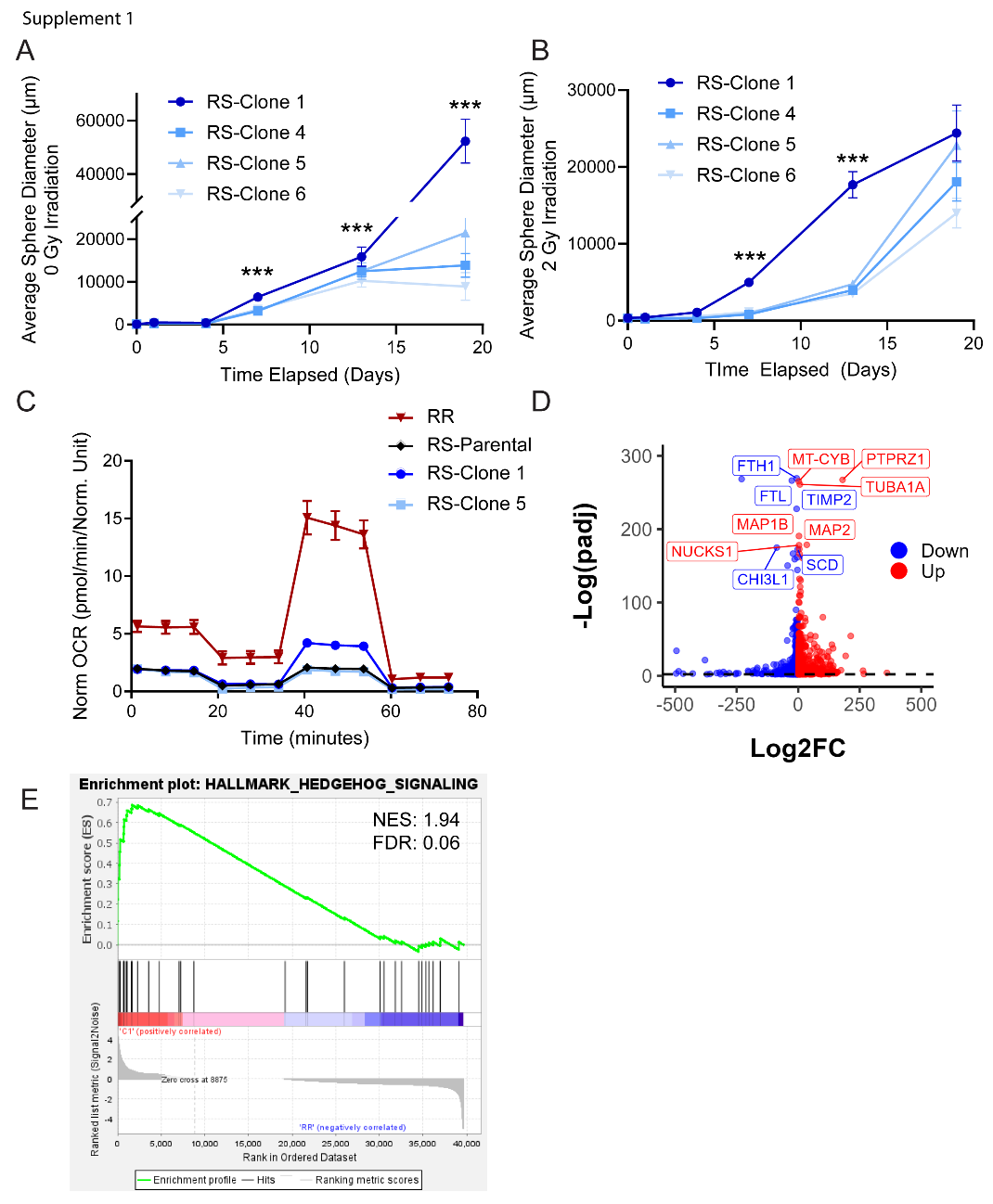


**Supplemental Figure 1: Growth and metabolic characteristics of RS-associated subclones.** (**A**) Colony formation assay showing the growth dynamics of RS-associated subclones under non-irradiated (0 Gy) conditions. Average sphere diameter was measured over time, n=3. (**B**) Colony formation assay of the same RS-associated subclones following 2 Gy irradiation, showing differential growth responses, n=3. (**C**) Mitochondrial stress test of RS-Parental, RR, and two RS-derived subclones using the Seahorse platform, n = 8. Data are presented as means ± SEM. Statistical significance was determined using two-way ANOVA followed by Tukey’s post-hoc test (**p* < 0.05, ***p* < 0.01, ****p* <0.001). All experiments were performed in biological triplicate. (D) Volcano Plot displaying differentially expressed genes in RS-Clone 1 versus RR (SF8628) bulk cell lines. Red points indicate significantly upregulated genes, blue points indicate downregulated genes (FDR <.01); horizontal dashed line denotes p value of .01. Log2 fold cutoffs are >2 and <-2. (E) Gene set enrichment analysis (GSEA) plot showing enriched Hedgehog signaling signatures in RS-Clone 1 vs RR bulk cell lines (NES = 1.94, FDR = 0.06).


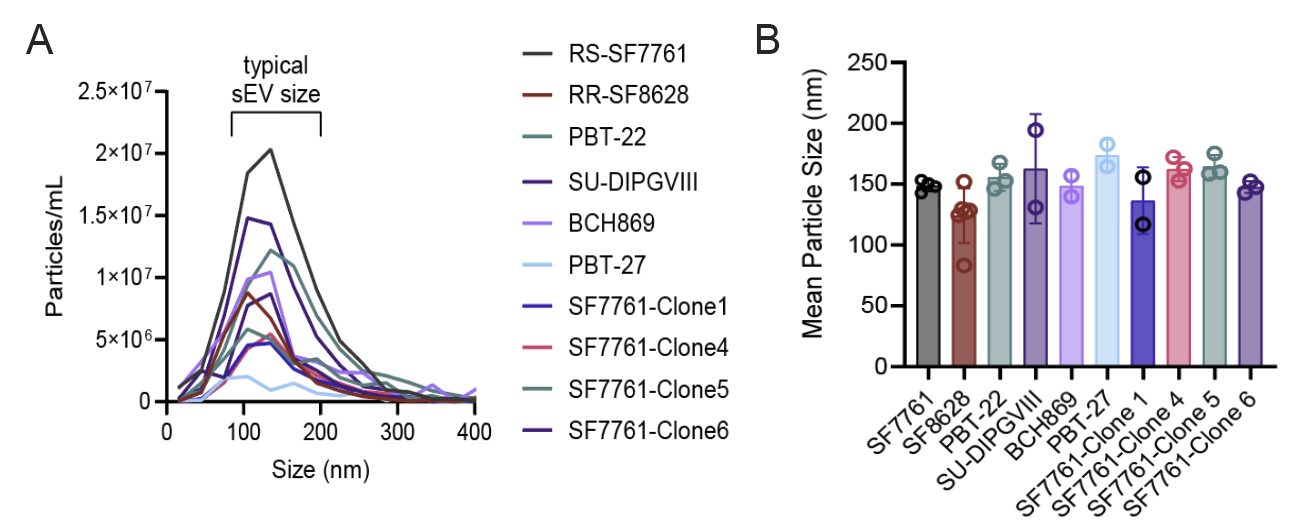


**Supplemental Figure 2: Characterization of small extracellular vesicles (sEVs) derived from H3K27M-pDMG cell lines and subclones.** (**A**) Nanoparticle tracking analysis (NTA) of sEVs isolated from multiple H3K27M-pDMG cell lines, including RS (SF7761), RR (SF8628), PBT-22, SU-DIPGVIII, BCH869, PBT-27, and four RS-derived subclones (SF7761-Clone 1, Clone 4, Clone 5, and Clone 6). The distribution of particle sizes is shown, with the typical sEV size range indicated. (**B**) Mean particle size of sEVs from each cell line, measured using the Zetaview system. Bars represent means ± standard error of the mean (SEM).


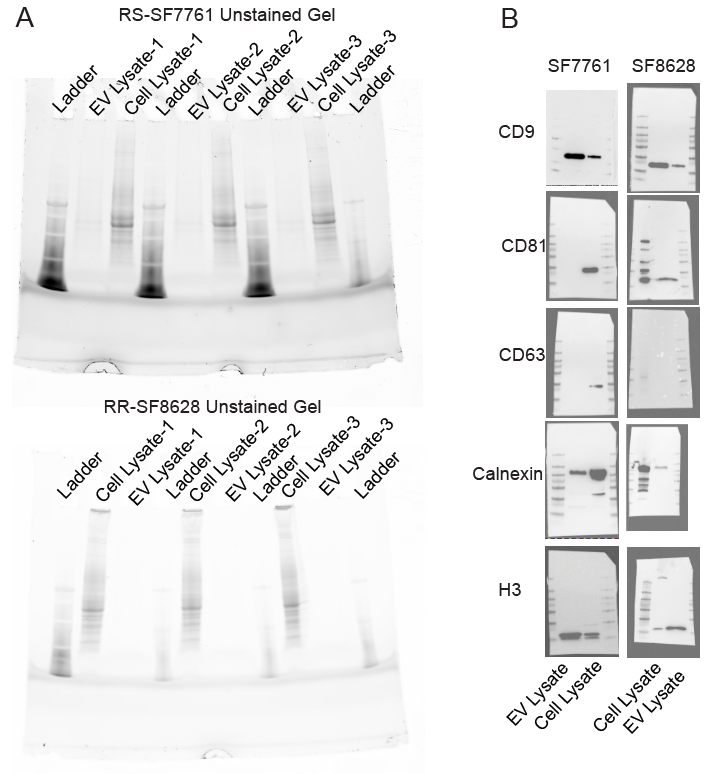


**Supplemental Figure 3: Total protein loading and blots for extracellular vesicle (EV) marker validation.** (**A**) Stain-free SDS-PAGE gel showing total protein loading of whole-cell lysates and extracellular vesicle (EV) lysates from RS (SF7761) and RR (SF8628) cells across three independent preparations. (**B**) Images of the blots corresponding to the cropped images in Figure 2, showing immunoblot detection of the indicated EV markers in whole-cell lysates (left lane) and EV fractions (right lane) from SF7761 and SF8628 cells.

**
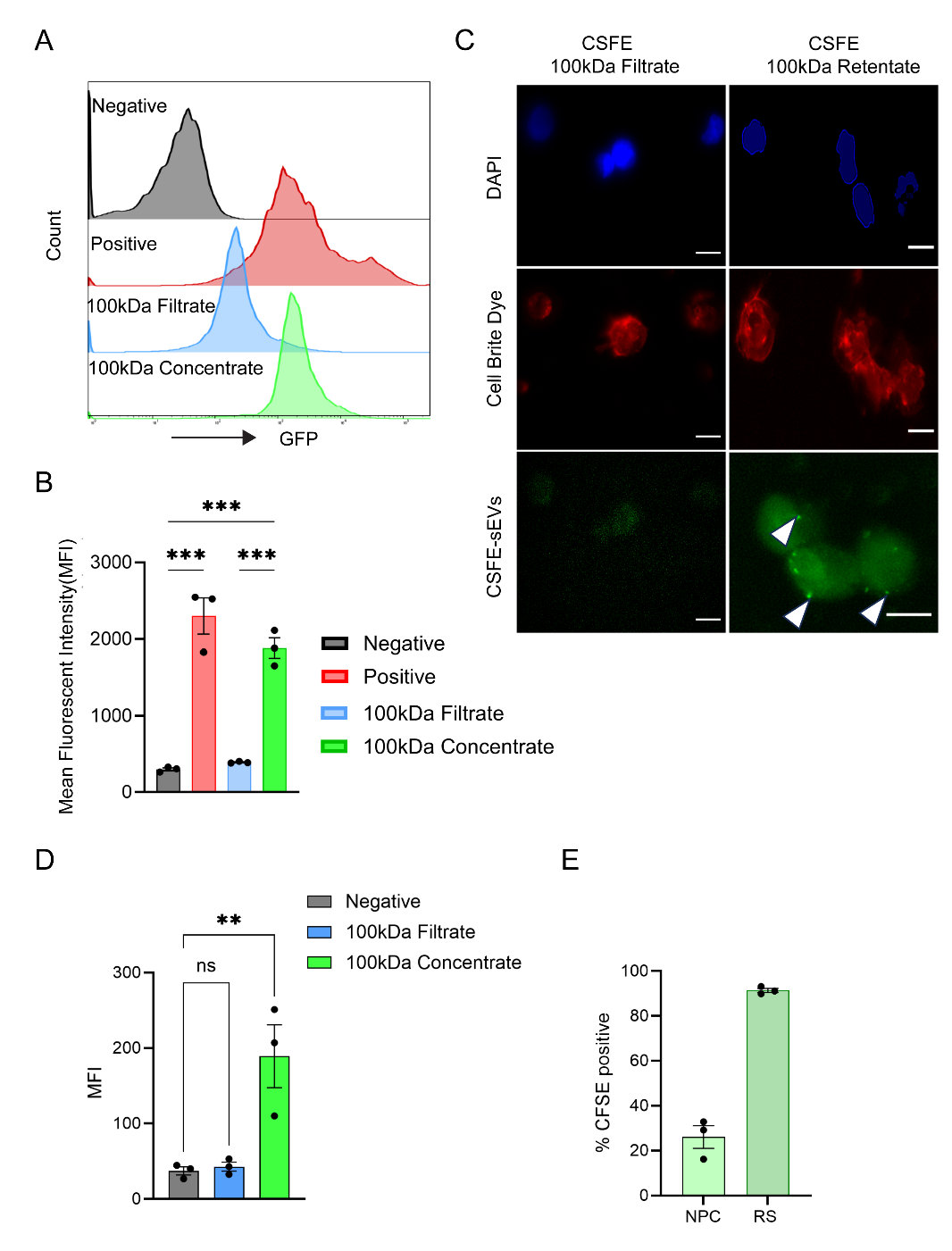
**

**Supplemental Figure 4: Small extracellular vesicle uptake assay** (**A**) Flow Cytometry profiles of 4 populations of RS cells, untreated (negative), cells stained with CSFE (positive), cells treated with 100 kDa filtrate of RR-sEVs dyed with CSFE (100 kDa filtrate), cells treated with 100 kDa concentrate of RR-sEVs dyed with CSFE (100 kDa concentrate). (**B**) MFI quantification of flow cytometry profiles in (A). (**C**) Representative images of high throughput immunofluorescent imaging of RS cells treated with RR-sEVs-CSFE stained with DAPI and Cell Brite. (D) MFI quantification of flow cytometry on NPCs after CSFE-RR-sEV 100kDa filtrate, concentrate, or negative control treatment for 2 hours. (E) Percent of CSFE positive cells compared to the entire population of cells quantified with Flow Cytometry after RR-sEV-CSFE 100kDa concentrate treatment for 2 hours on either a wildtype neural progenitor cell line (NPC) or RS cells. Statistical analysis performed in (B) and (D) was a 1-way Anova followed by a Tukey post-hoc. **p<.005,***p<.0001. All experiments were performed in biological triplicate.


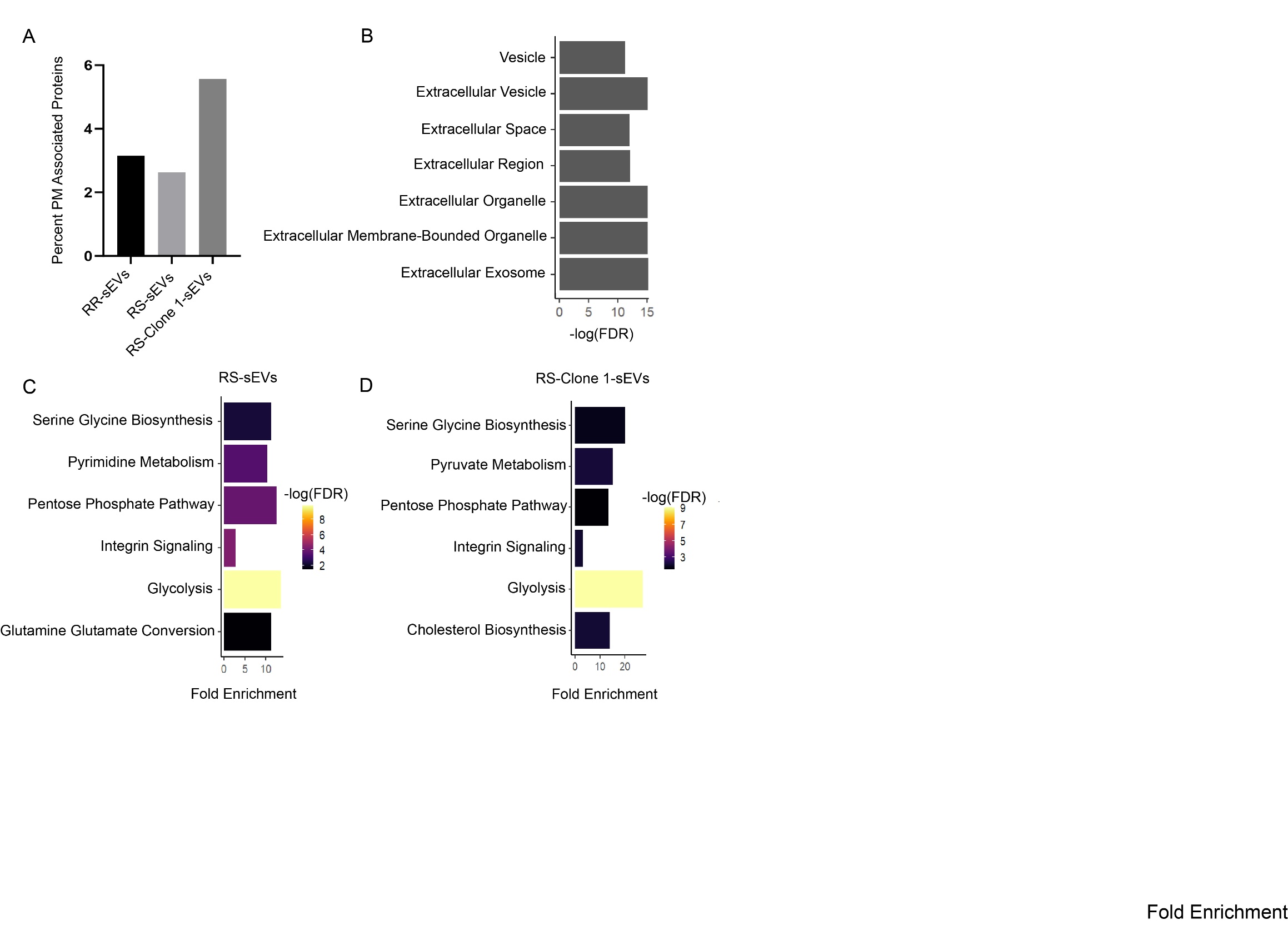


**Supplemental Figure 5: Proteomic pathway analysis of H3K27M-pDMG sEVs** (**A**) Percent of genes in sEVs from RR, RS, and RS-Clone 1 cells that are associated with the plasma membrane using the human proteome as a reference. (**B**) Top cellular components that represent the shared proteins among RR, RS, and RS-Clone 1 sEVs (**C**) - (**D**) Panther pathways that are represented by sEVs from RS, and RS-Clone 1 cells using a statistical overrepresentation analysis using a Fischer’s exact test. All pathway analysis was done using Panther. Cellular component analysis was done using G:profiler.


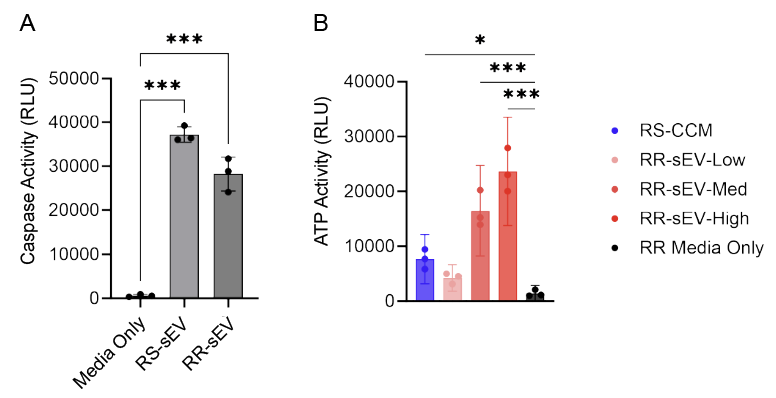


**Supplemental Figure 6: sEVs introduce confounding background signal in metabolic assays.** (**A**) Caspase-Glo 3/7 assay measuring luminescent caspase activity in media alone and after the addition of RS-sEVs or RR-sEVs. Both vesicle types induced high caspase signals (relative luminescence units, RLU) in the absence of cells, indicating potential assay interference. (**B**) CellTiter-Glo assay measuring ATP levels in media supplemented with increasing doses of RR-sEVs, RS-CCM, or RR media alone. RR-sEVs produced dose-dependent luminescent signal in the absence of cells, highlighting the limitations of this method for quantifying cell viability in the presence of sEVs. All experiments were performed in biological triplicate with at least two technical replicates per condition. Data are presented as means ± SEM. *p < 0.01, ***p < 0.0001 by one-way ANOVA with Tukey’s post-hoc test.


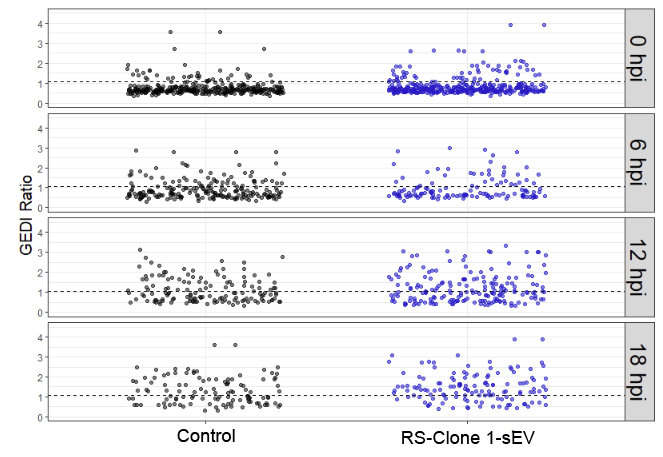


**Supplemental Figure 7: RS-Clone 1-sEVs improve survival of irradiated RS cells.** GEDI ratio values from RS cells stably expressing the GEDI construct, treated with RS-Clone 1-sEVs or control for 18 hours, followed by 8 Gy irradiation and live-cell imaging over 18 hours. Decreases in GEDI signal over time reflect death and detachment of cells from the plate. The experiment was performed with a seeding density of 2,500 cells per well, with at least two technical replicates per condition.


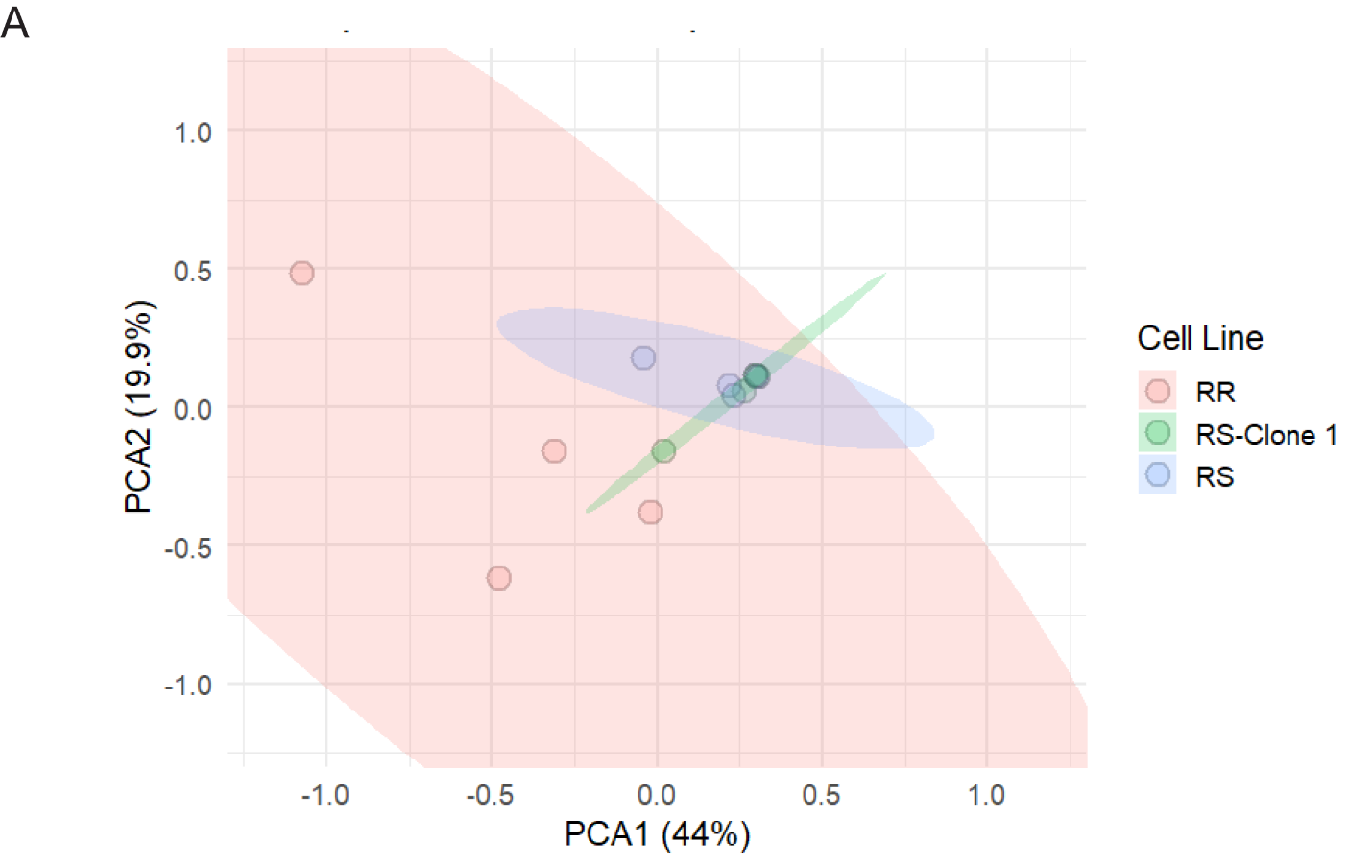


**Supplemental Figure 8: Unsupervised clustering of sEV small RNA sequencing from RR, RS-Clone 1, and RS cell lines. Multidimensional scaling of normalized read counts between RR, RS-Clone 1, and RS cell lines. Raw reads counts were processed using edgeR. Biological replicates for RR, RS Clone 1 and RS were 4, 5, and 3 respectively. Shaded ellipses represent 95% confidence intervals.**


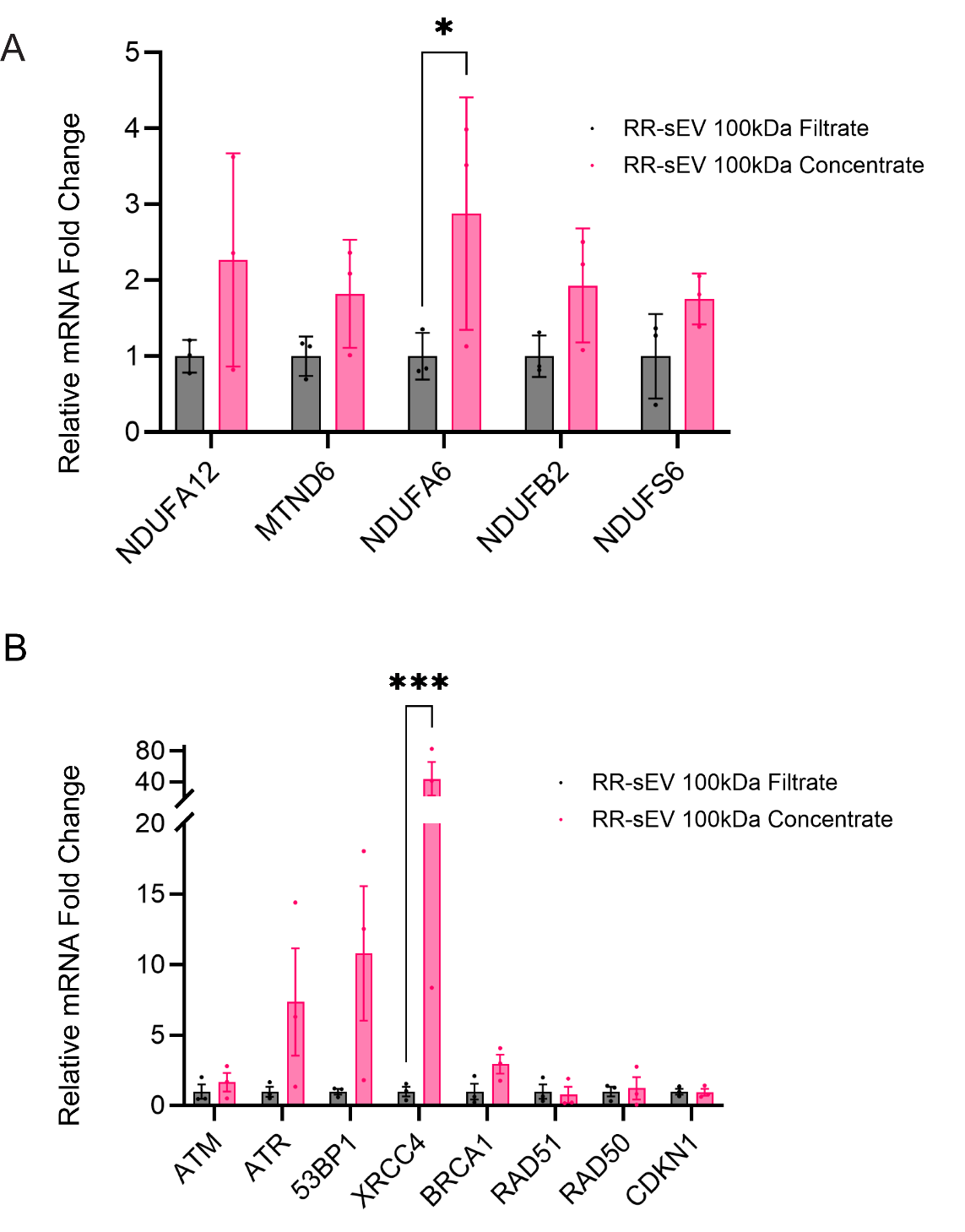


**Supplemental Figure 9: RR-sEV treated RS cells upregulate mitochondrial associated and DNA damage transcripts. Relative mRNA fold changes of RR-sEV treated RS cells from quantitative PCR on RS cells after 18 hours of RR-sEV or control treatment. (A) The top 5 upregulated genes from the bulk RNA-sequencing dataset in Figure 5 were validated for increased expression after RR-sEV treatment. (B) A panel of DNA damage response genes spanning general, NHEJ, and HR related DNA repair were tested for relative expression. Each gene had three technical replicates. There were 3 biological replicates/group and each was treated with a biologically different sEV preparation. Cells were dosed at 5000 sEVs/cell. *p<.05,***p<.0.0001. Error bars are standard error of the mean. 2-way ANOVA followed by a Sidak’s post-hoc was done to test for statistical significance between treatment groups for each gene.**
